# Vitamin D deficiency impairs skeletal muscle function in a smoking mouse model

**DOI:** 10.1530/JOE-15-0491

**Published:** 2016-05-01

**Authors:** Nele Cielen, Nele Heulens, Karen Maes, Geert Carmeliet, Chantal Mathieu, Wim Janssens, Ghislaine Gayan-Ramirez

**Affiliations:** 1 Laboratory of Respiratory DiseasesDepartment of Clinical and Experimental Medicine, KULeuven, Leuven, Belgium; 2 Laboratory of Clinical and Experimental EndocrinologyDepartment of Clinical and Experimental Medicine, KULeuven, Leuven, Belgium

**Keywords:** COPD, muscle function, muscle atrophy, oxidative stress, cigarette smoke exposure, lung function

## Abstract

Chronic obstructive pulmonary disease (COPD) is associated with skeletal muscle dysfunction. Vitamin D plays an important role in muscle strength and performance in healthy individuals. Vitamin D deficiency is highly prevalent in COPD, but its role in skeletal muscle dysfunction remains unclear. We examined the time-course effect of vitamin D deficiency on limb muscle function in mice with normal or deficient vitamin D serum levels exposed to air or cigarette smoke for 6, 12 or 18 weeks. The synergy of smoking and vitamin D deficiency increased lung inflammation and lung compliance from 6 weeks on with highest emphysema scores observed at 18 weeks. Smoking reduced body and muscle mass of the soleus and extensor digitorum longus (EDL), but did not affect contractility, despite type II atrophy. Vitamin D deficiency did not alter muscle mass but reduced muscle force over time, downregulated vitamin D receptor expression, and increased muscle lipid peroxidation but did not alter actin and myosin expression, fiber dimensions or twitch relaxation time. The combined effect of smoking and vitamin D deficiency did not further deteriorate muscle function but worsened soleus mass loss and EDL fiber atrophy at 18 weeks. We conclude that the synergy of smoking and vitamin D deficiency in contrast to its effect on lung disease, had different, independent but important noxious effects on skeletal muscles in a mouse model of mild COPD.

## Introduction

Chronic obstructive pulmonary disease (COPD) is not only a lung disease, but also associates with several comorbidities. Among them, skeletal muscle dysfunction is of major concern, as it contributes, independently of lung function (Engelen *et al.* 2000), to decreased functional capacity (Gosselink *et al.* 1996), poor quality of life (Simpson *et al.* 1992), increased health care utilization (Decramer *et al.* 1997), and even mortality (Swallow *et al.* 2007). In addition to smoking, physical inactivity, oxidative stress, and systemic inflammation, other factors such as vitamin D deficiency may contribute to the large variability in the prevalence and severity of skeletal muscle dysfunction in COPD (Maltais *et al.* 2014).

Low serum levels of vitamin D are known to be associated with reduced muscle strength and performance, and lead to muscle atrophy, increased apoptosis, decreased protein synthesis, and perturbation in intracellular calcium homeostasis (Ceglia & Harris 2013). Most of these alterations are also observed in the skeletal muscles of patients with COPD (Agusti *et al.* 2002, Doucet *et al.* 2007, Plant *et al.* 2010). In addition, vitamin D deficiency, defined as serum levels of 25-hydroxyvitamin D (25-(OH)D) below 20 ng/ml (50 nmol/l), is highly prevalent in patients with COPD and increases with the severity of the disease (Janssens *et al.* 2009, 2010). However, the association between vitamin D serum levels and muscle strength, as proven in controls, was not observed in COPD patients (Jackson *et al.* 2013), suggesting that some patients may represent resistance to the actions of vitamin D (Jackson *et al.* 2013). Interestingly, a relationship between variants of the vitamin D receptor (VDR) and skeletal muscle strength was observed in patients with COPD (Hopkinson *et al.* 2008).

Although the involvement of vitamin D deficiency on skeletal muscle dysfunction in patients with COPD is unclear, several studies suggest a potential link. First, the high levels of parathyroid hormone as observed in patients with COPD (Jackson *et al.* 2013) may enhance the risk of sarcopenia (Visser *et al.* 2003) and accentuate an age-related reduction in VDR expression (Bischoff-Ferrari *et al.* 2004*a*) and muscle strength (Bischoff-Ferrari *et al.* 2004*b*). Furthermore, cigarette smoke exposure has been shown to increase the risk for vitamin D deficiency (Cutillas-Marco *et al.* 2012) which may enhance the risk for sarcopenia by impairing muscle protein synthesis (Petersen *et al.* 2007). In mice, smoking downregulates PGC1-α (Tang *et al.* 2010) which serves as a co-activator for VDR (Savkur *et al.* 2005) thereby affecting skeletal muscle function. Apart from hypocalcemia, hypophosphatemia, and secondary hyperparathyroidism (Li *et al.* 1997, Yoshizawa *et al.* 1997, Song *et al.* 2003), *Vdr-*knockout mice also exhibited generalized fiber atrophy and alterations in myogenic differentiation pathway (Endo *et al.* 2003). Finally, in cell culture studies, cigarette smoke is found to inhibit vitamin D induced-VDR translocation (Uh *et al.* 2012) and to impair several of the vitamin D actions on skeletal muscle cells (Liu *et al.* 2011).

To investigate whether vitamin D deficiency *per se* may play a role in skeletal muscle dysfunction in COPD, we examined the time-course effect of chronic vitamin D deficiency on skeletal muscle function in a smoking mouse model. We used a vitamin D-deficient mouse model with normalized calcium and phosphorus serum levels by giving mice a rescue diet from gestation onward (Johnson & DeLuca 2002) and exposed them to cigarette smoke or room air for 6, 12, or 18 weeks. We hypothesized that vitamin D deficiency *per se *would exert detrimental effects on skeletal muscle, which would be potentiated when combined with smoking.

## Materials and methods

### Animals

Vitamin D deficiency was induced in C57Bl/6 mice by keeping them in an ultraviolet (UV) light-free environment and by feeding them a vitamin D-depleted diet (<200 IU/kg vitamin D) containing 20% w/w lactose, 2% w/w calcium, and 1.25% w/w phosphorus (Ssniff Bio-services, Uden, The Netherlands) to maintain normal calcium and phosphorus serum levels (Johnson & DeLuca 2002). At the age of 8 weeks, breeding pairs were formed and their male offspring were used in the study. During the whole-study experiment, these mice were kept in an UV light-free environment and received the vitamin-D depleted diet. Male C57Bl/6 mice from the same age were obtained from Harlan (Horst, The Netherlands) and used as controls. They were fed with a normal diet (Ssniff) containing 1000 IU/kg vitamin D to maintain the daily requirement of vitamin D. All experimental procedures were approved by the Ethical Committee of Animal Experiments by the KULeuven.

### Study design

At the age of 8 weeks, vitamin D-deficient mice (24.4±2.0 g) were daily exposed to breathe ambient air (D-Air, *n*=42) or cigarette smoke (D-Smoking, *n*=40) via a nose-only exposure system (InExpose system, Scireq, Paris, France). After a short acclimatization period, smoking mice were exposed to four cigarettes (Kentucky Tobacco Research and Development Center, University of Kentucky, Lexington, KY, USA), two times a day, 5 days a week (Rinaldi *et al.* 2012) for a period of 6, 12, or 18 weeks. They were compared with mice with sufficient vitamin D levels (26.7±0.9 g) exposed to ambient air (N-Air, *n*=36) or to cigarette smoke (N-Smoking, *n*=36) for the same duration (for details on number of mice per group, see Supplementary Table 1, see section on supplementary data at the end of this article). Body mass was measured weekly and total particle density concentration was measured daily (Microdust, Casella CEL, Bedford, UK). Total particle density averaged 754 mg per m^3^.

### Pulmonary function measurements

Twenty-four hours after the last exposure to cigarette smoke or ambient air, mice were intraperitoneally anesthetized with a mixture of xylazine (8.5mg/kg, Rompun, Bayer) and ketamine (130mg/kg, Ketalar, Pfizer). After tracheotomy was performed with a standard catheter (CNS5002, Buxco, Wilmongton, NC, USA), mice were placed in a whole-body plethysmograph with the tracheotomy catheter being connected to a computer-controlled ventilator (Buxco-Force Pulmonary Maneuvers). After a regular breathing pattern was obtained, the quasi-static pressure volume maneuver is started. This maneuver inspires the animal to total lung capacity, hold that pressure for a short period of time, and then slowly expires the animal to residual volume (De Vleeschauwer *et al.* 2011, Rinaldi *et al.* 2012). Lung compliance is calculated from the pressure–volume curve, and an average of three signals was used for subsequent analysis.

### Broncho-alveolar lavage

Lungs were lavaged four times with 1 ml of Dulbecco’s phosphate-buffered saline (PBS) and centrifuged at 1000***g ***for 10 min at 4°C). Cytospins were colored with May–Giemsa staining to determine differential cell counts. Three hundred cells per mouse were counted to determine the number of neutrophils.

### Histopathology of the lungs

The left lung was fixed in 6% v/v paraformaldehyde at a constant hydrostatic pressure of 25 cm H_2_O for 24 h and embedded in paraffin. Sagittal sections were stained with hematoxylin and eosin to evaluate airspace enlargement quantified by mean linear intercept. The mean linear intercept was measured in 15 randomly selected fields per slide at a 200× magnification and calculated as the total length of the grid lines×random fields divided by the sum of the alveolar intercepts (Dunnill 1964).

### Serum measurements

Blood was drawn from the vena cava and centrifuged at 9300***g*** for 10 min at 4°C. 25-(OH)D serum levels were measured by liquid-phase radioimmunoassay.

### Muscle mass

The soleus, extensor digitorum longus (EDL), and gastrocnemius muscles from both hindlimbs were weighed and mass of left and right muscles was summed and presented in the Results section.

### *In vitro* muscle contractile properties

*In vitro *contractile properties were assessed in the left soleus and EDL muscles, as described previously (Matuszczak *et al.* 2004, Rinaldi *et al.* 2012). Briefly, contractility was measured *in vitro* at 37°C using a temperature-controlled organ bath and stimulating electrodes. Optimal muscle length (Lo) for peak twitch force was established and the following measurements were performed at Lo: (i) maximum twitch force, (ii) maximum tetanic force (300 Hz), and (iii) force–frequency relationship at 1, 30, 50, 80, 150, and 250Hz. Twitch half-relaxation time was calculated. Lo was measured as well as bundle weight. Absolute (in gram) and specific (normalized for cross-sectional area) forces were used for analysis. Cross-sectional area was calculated by muscle weight divided by specific density (1.056 g/cm^2^) and muscle Lo.

### Muscle histology

Right soleus and EDL muscles were frozen in isopentane cooled by liquid nitrogen and stored at −80°C. To determine the dimensions and proportions of the different muscle fibers, serial cross sections of 5μm thickness were blocked (10% v/v normal goat serum in PBS) for 1 h. Afterwards, the slides were incubated at room temperature for 2 h with a primary antibody cocktail, specific to laminin (ab11575, Abcam), myosin heavy chain (MHC)-1 (BA-F8), MHC2A (SC-71), and MHC2B (BF-F3) in the blocking buffer. All MHC antibodies were purchased from Developmental Studies Hybridoma Bank (Iowa City, IA, USA). After washing, the slides were incubated for 1h with a secondary antibody cocktail (Alexa Fluor 532, Alexa Fluor 350, Alexa Fluor 488, and Alexa Fluor 555, Life Technologies) in the blocking buffer. The sections were mounted with cover slips with ProLong Gold antifade reagent (Life Technologies). Using a computerized image system (CellSens, Olympus), about 150 fibers per muscle were used to determine the dimensions and proportions of the different fibers.

### Myosin-to-actin ratio

Myosin-to-actin ratio was measured at 18weeks in the gastrocnemius muscle. Muscle samples (±40mg) were homogenized using a glass homogenizer in 1 ml of a 250 mmol/L sucrose buffer (in mmol/L: 250 sucrose, 100 KCl, five EDTA, 20 imidazole, pH 6.8) on ice (Hasten *et al.* 1998). The homogenate was centrifuged at 1200***g*** for 10 min, and the supernatant was discarded. The pellet was suspended in 1 ml of a 0.5% Triton X-100 solution (175 mmol/L KCl, 0.5% v/v Triton X-100, pH 6.8). The suspension was homogenized and centrifuged as described before. The resultant pellet was rinsed with 1 ml of wash buffer (150 mmol/L KCl, 20 mmol/L Tris, pH 7.0). After centrifugation, the pellet was suspended in 200 μl of wash buffer. Protein concentration was measured according to Bradford method. Proteins were separated on an 8% v/v polyacrylamide gel. Myosin and actin were detected using Coomassie blue staining and semi-quantified using a computerized image system (Bio1D, VilberLourmat, Marne-La-Vallée, France).

### VDR expression

VDR expression of the soleus and EDL muscles was measured by immuno staining at 18 weeks. Muscle sections of 5 μm thickness were simultaneously fixed and permeabilized by incubation in 3% v/v paraformaldehyde and 0.1% v/v Triton X-100 in PBS for 30 min (Girgis *et al.* 2014). After thorough washing in PBS, sections were then blocked (10% v/v normal goat serum in PBS) for 30 min and incubated with a mouse monoclonal VDR antibody (sc-13133, SantaCruz Biotechnology) in the blocking buffer at 4°C overnight. Sections were then blocked for 30 min at room temperature and subsequently incubated for 1 h in PBS with secondary antibody (Alexa Fluor 488, Life Technologies). Afterwards, sections were incubated with 4′,6-diamidino-2-phenylindole (Life Technologies) for 5 min to stain nuclei. The sections were mounted with cover slips with ProLong Gold antifade reagent. Images were taken using a computerized image system (CellSens, Olympus). VDR expression was blindly quantified as the number of positive spots per 150 muscle fibers.

### Oxidative stress measurement

During the lipid peroxidation cascade, the primary adduct that is formed is 4-hydroxy-2-nonenal (4HNE). Using immunoblotting, 4HNE was measured in the gastrocnemius muscle as an index of lipid peroxidation. Therefore, frozen gastrocnemius samples (±10 mg) were homogenized in a buffer containing 5 mM Tris–HCl and 5 mM EDTA (pH7.5). The homogenates were centrifuged at 4°C for 20 min at 10,000***g***. After centrifugation, supernatant was collected and protein content was determined by Bradford method. Proteins (20μg per lane) were separated on a 7.5% SDS–polyacrylamide gel and blotted onto a polyvinylidene difluoride membrane. Afterwards, the blots were incubated overnight at 4°C with a monoclonal mouse anti 4-HNE antibody (MAB3249, R&D Systems) and subsequently with a polyclonal rabbit anti-mouse secondary antibody (p0260, DAKO) for 1h at room temperature. Proteins were detected using chemiluminescent peroxidase substrate (according to the manufacturer’s instructions, Amersham) and imaged with the Proxima 2850T imaging system (Isogen Life Technologies, De Meern, The Netherlands). Four bands per sample were quantified using the TotalLab 1D (Isogen Life Technologies).

### Statistical analyses

Statistical analyses were performed using a SAS 9.3 Statistical Package (SAS Institute, Cary, NC, USA). To test for normality, the Shapiro–Wilk test was applied. Comparisons between the four groups of animals were performed using two-way ANOVA with the inclusion of an interaction term between smoking and vitamin D serum levels, followed by a Tukey’s *post hoc* test. *P* values <0.05 were considered to be statistically significant. Data are expressed as mean±s.d.

## Results

### Serum measurements

The vitamin D serum levels (25-(OH)D) were 75% lower in the vitamin D-deficient mice (18±7ng/ml) compared with the mice with normal vitamin D levels (77±13ng/ml; *P*<0.0001).

### Pulmonary disease

**Figure 1:**
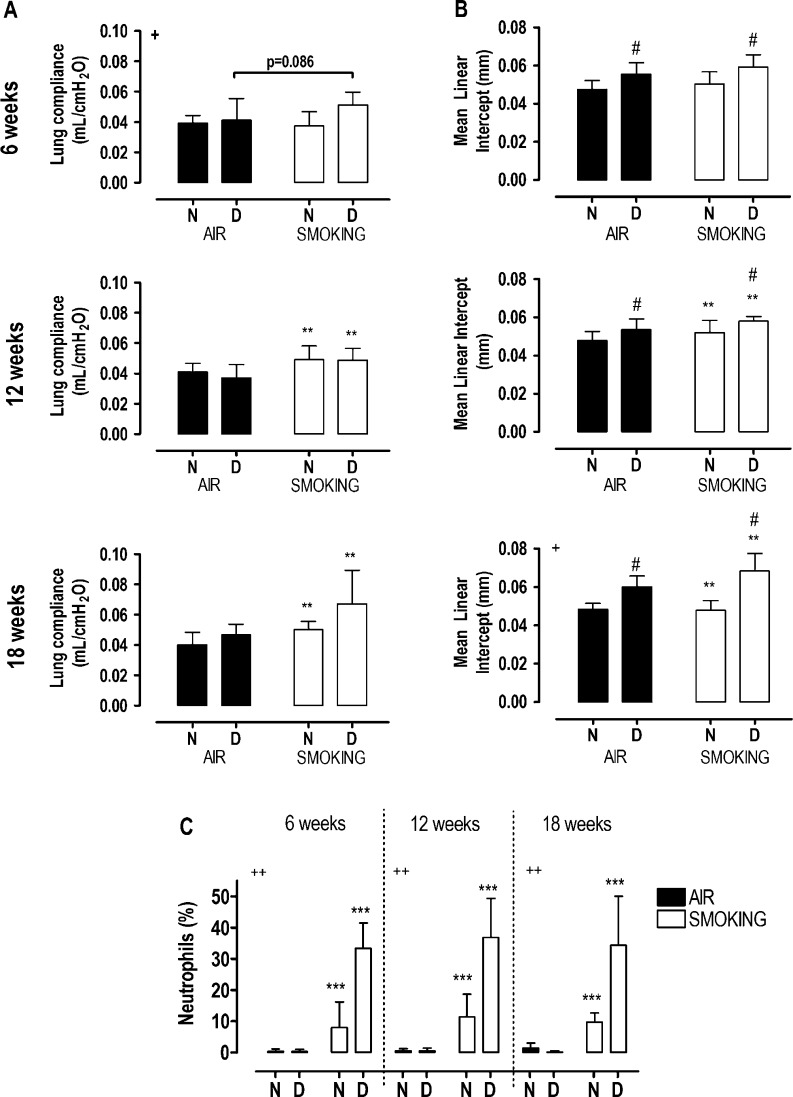
Lung compliance (A), mean linear intercept (B) and neutrophils (C) in broncho-alveolar lavage fluid at 6, 12, and 18 weeks in mice with normal (N) or deficient (D) levels of vitamin D, breathing room air (black bars) or exposed to cigarette smoke (white bars). Values are mean±s.d. **P<0.001 and ***P<0.0001: effect of smoking; #*P*<0.01 effect of vitamin D deficiency. Interaction between vitamin D deficiency and smoking (+*P*<0.05 and ++*P*<0.0001).

A trend toward increased lung compliance (+24%) was observed after 6 weeks of smoking in the vitamin D-deficient mice compared with vitamin D-deficient mice breathing air (*P*=0.086, interaction term *P*<0.05), while after 12 and 18 weeks, lung compliance was enhanced with smoking (*P*<0.001), with no additional effect of vitamin D deficiency (Fig. 1A). Mean linear intercept increased similarly after 12 and 18 weeks of smoking (+7%, *P*<0.05), and was higher at each time point in vitamin D deficiency groups (*P*<0.01). Moreover, a synergic increase in mean linear intercept was observed after 18 weeks when vitamin D deficiency was combined with smoking (interaction term *P*=0.02; Fig. 1B and Supplementary Figure 1, see section on supplementary data at the end of this article). Smoking caused a shift toward increased number of neutrophils at each time point (*P*<0.0001), and this effect was more pronounced in vitamin D-deficient smoking mice (interaction term *P*<0.0001; Fig. 1C). In addition, the number of macrophages reduced with smoking at all time points (−8%, pooled values), and this reduction was also more pronounced with vitamin D deficiency (−40%, pooled values). Finally, number of lymphocytes increased only when vitamin D deficiency was combined with smoking (+5%, pooled values; data not shown).

### Body and muscle mass

**Figure 2:**
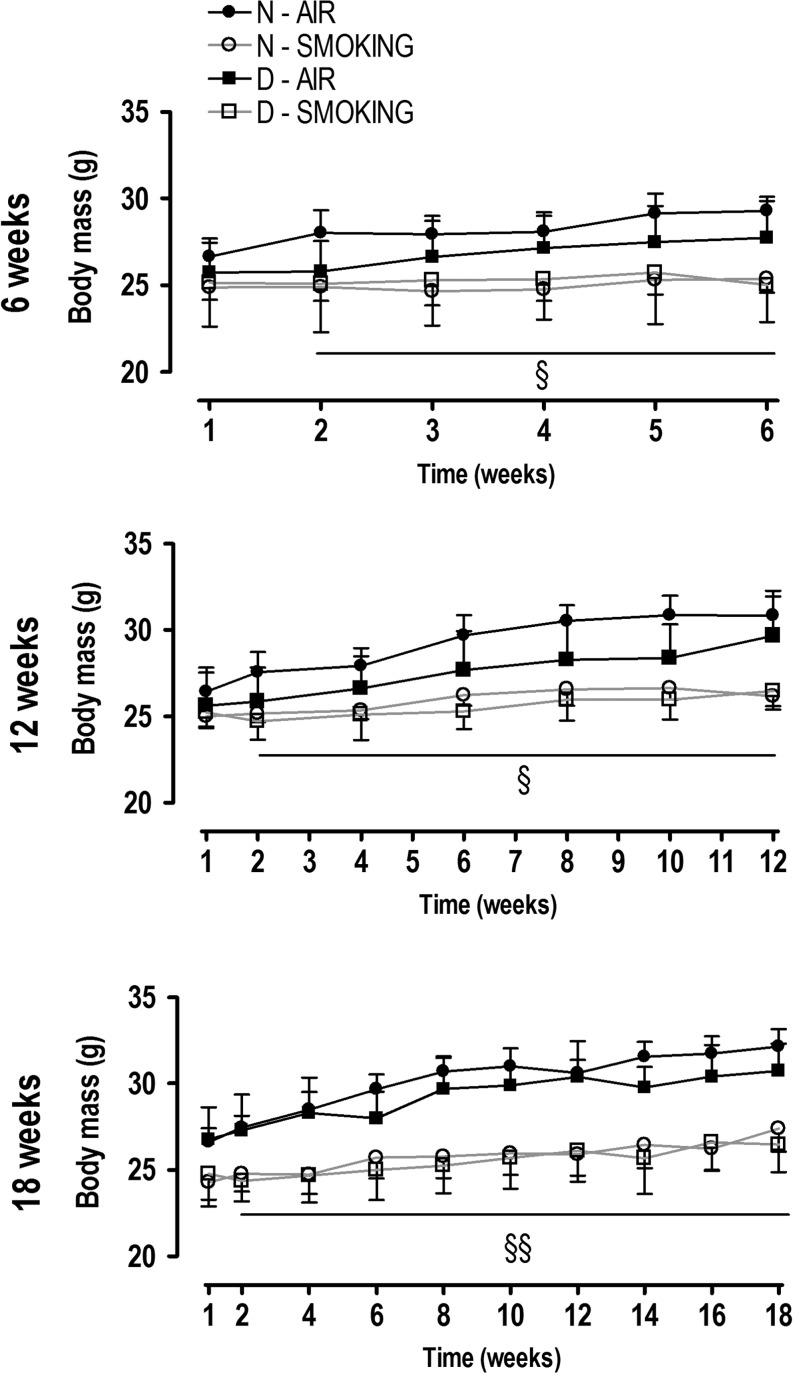
Body mass evolution after 6, 12, and 18 weeks in mice with normal (N, circles) or deficient (D, squares) levels of vitamin D, breathing room air (closed symbols) or exposed to cigarette smoke (open symbols). Values are mean±s.d. Body mass evolution with interaction between time and body mass (two way ANOVA and *post hoc*): §*P*<0.05 smokers vs N-air and §§*P*<0.0001 smokers vs air.

**Figure 3:**
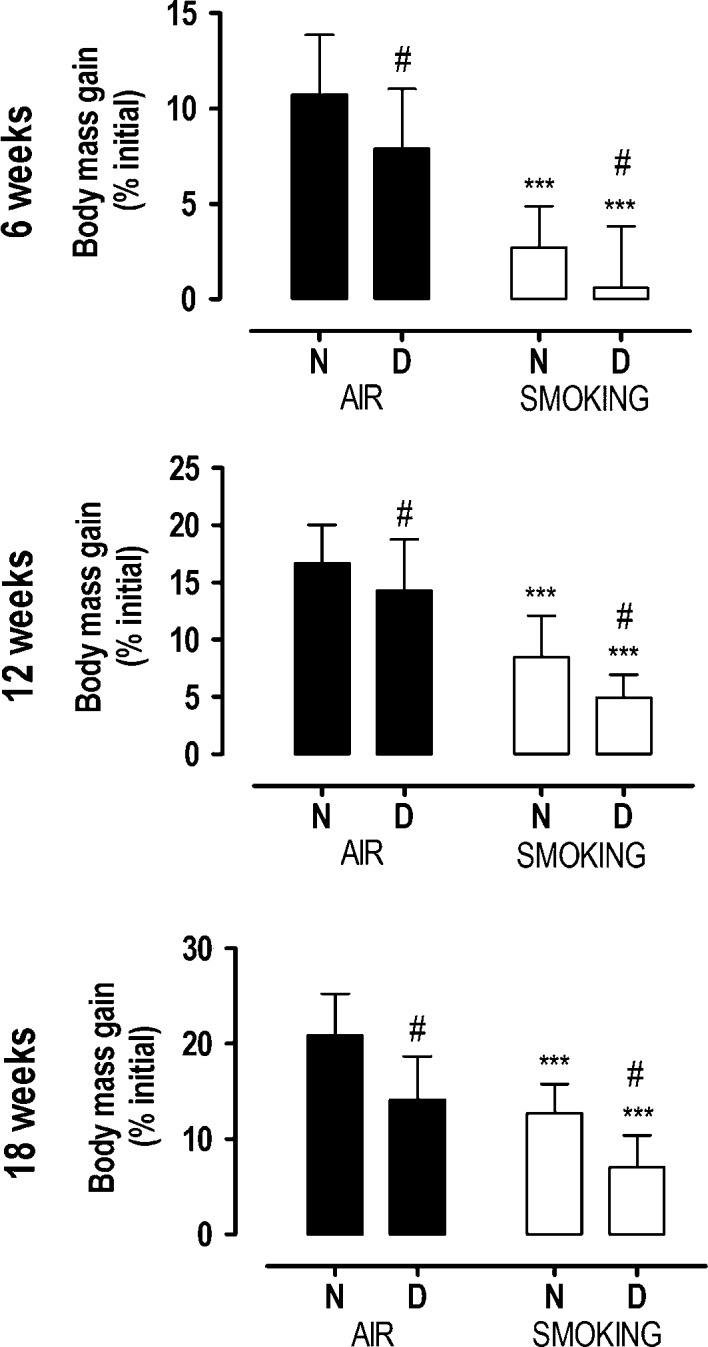
Body mass gain after 6, 12, and 18 weeks in mice with normal (N) or deficient (D) levels of vitamin D, breathing room air (black bars) or exposed to cigarette smoke (white bars). Values are mean ± s.d. ***P < 0.0001 effect of smoking; #*P* < 0.01 effect of vitamin D deficiency. There is no interaction between vitamin D deficiency and smoking.

Starting body mass (week 0) was lower in vitamin D-deficient mice (24.4±2.0g) compared with mice with normal vitamin D levels (26.7±0.9g; *P*<0.0001). All mice gradually increased their body mass, but from the second week already, mice in smoking condition exhibited a reduced mass gain (Fig. 2) compared with those in nonsmoking condition (interaction term *P*<0.05). Moreover, at each time point, body mass gain was lower in vitamin D-deficient mice compared with the non-deficient groups (Fig. 3; *P*<0.01), which resulted in the lowest body mass gain in vitamin D-deficient smoking mice at every single time point. Despite the independent effect of smoking and vitamin D deficiency on body mass gain, there was no significant statistical interaction (*P*>0.4).

While vitamin D deficiency did not alter muscle mass, smoking reduced EDL mass (−6 to −13%), gastrocnemius mass (−7 to −13%), and soleus mass (−9 to −15%) at 6, 12, and 18 weeks compared with air-breathing mice (Table 1). At 18 weeks of smoking, loss of soleus mass was highest only when vitamin D deficiency was combined with smoking, compared with mice with normal vitamin D levels (−20% vs −10%, interaction term *P*=0.0295; Table 1).

**Table 1:** Muscle mass (mg) of extensor digitorum longus (EDL), soleus (SO), and gastrocnemius (GA) muscles. Muscle mass in mice with normal or deficient vitamin D levels, breathing room air (air) or exposed to cigarette smoke (smoking) during 6, 12, and 18 weeks. Values expressed as the sum of left and right muscle mass. Values are mean ± s.d.

	**Air**		**Smoking**		**Interaction term**
	**Normal**	**Deficient**	**Normal**	**Deficient**	
6 weeks					
EDL	22.0±1.1	23.7±2.5	20.8±1.4^a^	22.5±2.0^a^	
SO	20.5±1.9	22.8±1.7	18.9±1.2^a^	20.3±2.4^a^	
GA	262.9±21.9	280.8±18.0	240.3±13.1^a^	262.8±21.0^a^	
12 weeks					
EDL	22.9±1.3	25.3±2.0	20.9±1.7^b^	23.5±1.3^b^	
SO	21.3±1.9	23.6±2.1	19.8±1.7^a^	21.4±1.5^a^	
GA	267.4±16.5	285.7±20.8	245.7±23.1^b^	258.5±18.4^b^	
18 weeks					
EDL	24.6±2.2	26.9±2.0	21.6±1.6^c^	23.5±2.4^c^	
SO	22.6±2.0	24.7±2.2	20.4±1.6^c^	19.7±2.2^c^	^d^
GA	281.9±22.7	282.0±18.6	242.5±15.3^c^	249.0±18.2^c^	

^a^*P* < 0.05, ^b^*P* < 0.001 and ^c^*P* < 0.0001 effect of smoking. Interaction between vitamin D deficiency and smoking (^d^*P* = 0.0295).

### Muscle histology

**Figure 4:**
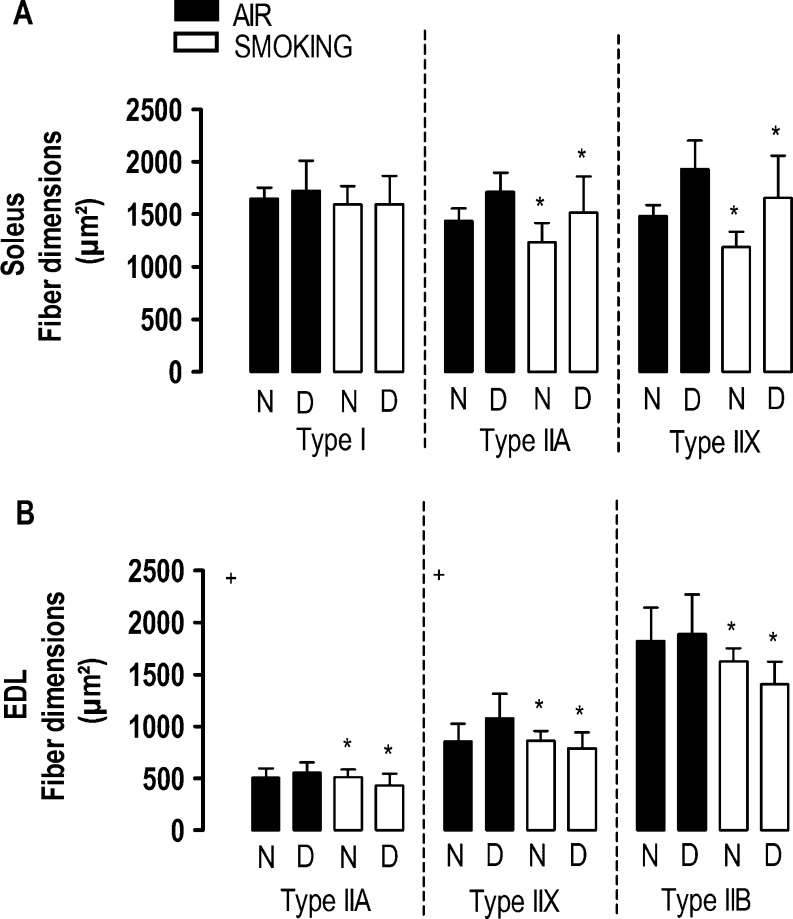
Fiber dimensions of the soleus (A) and EDL (B) muscle after 18 weeks in mice with normal (N) or deficient (D) vitamin D levels, breathing room air (black bars) or exposed to cigarette smoke (white bars). Values are mean ± s.d. *P<0.05 effect of smoking. Interaction between vitamin D deficiency and smoking (+*P*<0.05).

Vitamin D deficiency did not alter fiber proportions or fiber type dimensions of either muscles at any time point. Although smoking did not affect fiber proportions, it reduced the dimensions of all the type II fibers of the soleus muscle at 12 and 18 weeks (Fig. 4A) and of EDL muscle at 18 weeks (*P*<0.05; Fig. 4B and Supplementary Figure 2). In the EDL muscle, an interaction term between vitamin D deficiency and smoking was observed in atrophy of the type IIa (interaction term *P*=0.0487) and the type IIx fibers (interaction term *P*=0.0174) after 18 weeks.

### Muscle contractile properties

**Figure 5:**
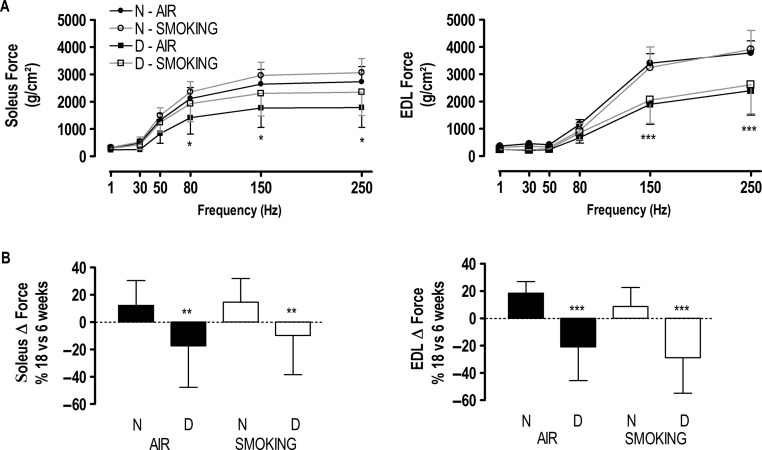
Force–frequency curve (A) of the soleus and extensor digitorum longus (EDL) muscles at 18 weeks in mice with normal (circles) or deficient (squares) vitamin D levels breathing room air (closed symbols) or exposed to cigarette smoke (open symbols). Values are mean±s.d. Interaction between frequency and force (two way ANOVA and *post hoc*): *P<0.05 D-air vs N; ***P<0.0001 deficient vs normal. Maximal tetanic force differences (Δ) (B) of the soleus and EDL muscles between 18 and 6 weeks in mice with normal (N) or deficient (D) vitamin D levels, breathing room air (black bars) or exposed to cigarette smoke (white bars). Values are mean±s.d. and expressed as percentage difference between 18 and 6 weeks. **P<0.01 and ***P<0.0001 effect of vitamin D deficiency. There is no interaction between vitamin D deficiency and smoking.

Despite its effects on muscle mass and fiber type dimensions, smoking did not affect contractility of both muscles at any time point, even when muscle force was expressed in absolute values (not corrected for cross-sectional area; Table 2). This was also the case for vitamin D deficiency at 6 weeks. However, after 12 and 18 weeks, specific but not absolute force of the soleus muscle was lower at high stimulation frequencies in vitamin D-deficient mice breathing room air (Fig. 5A and Table 2). This was similar for the EDL muscle at 12 weeks (interaction term *P*=0.0249) while EDL absolute and specific forces were both reduced at 18 weeks in vitamin D-deficient groups (*P*<0.0001; Fig. 5A and Table 2) in both smoking and air-exposed conditions. When looking at the loss of contractility by comparing specific force generated at 18 weeks with that at 6 weeks, a significant drop in both soleus and EDL forces was observed in all vitamin D-deficient conditions, which contrasted with the moderate gain in specific muscle force in smoking mice (Fig. 5B). Overall, we found no significant interaction between smoking and vitamin D deficiency on muscle force in either the EDL or soleus muscle. Neither smoking nor vitamin D deficiency affected twitch half-relaxation time (data not shown).

**Table 2:** Maximal tetanic force in absolute (g) and specific (g/cm^2^) values of the extensor digitorum longus (EDL) and soleus (SO) muscles. Maximal tetanic force in mice with normal or deficient vitamin D levels, breathing room air (air) or exposed to cigarette smoke (smoking) during 6, 12, and 18 weeks. Values are expressed as mean±s.d.

	**Air**				**Smoking**				**Interaction term**
	**Normal**		**Deficient**		**Normal**		**Deficient**		
	**Absolute**	**Specific**	**Absolute**	**Specific**	**Absolute**	**Specific**	**Absolute**	**Specific**	
6 weeks									
EDL	37±5	4156±773	37±4	3748±597	35±6	4252±819	41±3	4520±374	
SO	21±3	2769±533	24±5	2567±735	22±4	2778±524	24±5	2877±508	
12 weeks									
EDL	38±8	4609±907	33±6	3393±757^a^	35±5	4303±479	38±4	4182±740^a^	^c^
SO	28±3	3742±348	26±5	2850±494^b^	25±4	3654±512	25±6	3095±759^b^	
18 weeks									
EDL	43±4	4926±353	30±9^b^	2967±929^b^	38±3	4618±596	30±10^b^	3216±1175^b^	
SO	25±3	3111±505	21±8	2124±782^a^	24±4	3183±486	22±7	2595±822^a^	

^a^*P*<0.01 and ^b^*P*<0.0001 effect of vitamin D deficiency. Interaction term between vitamin D deficiency and smoking (^c^*P*=0.0249).

### VDR expression

**Figure 6:**
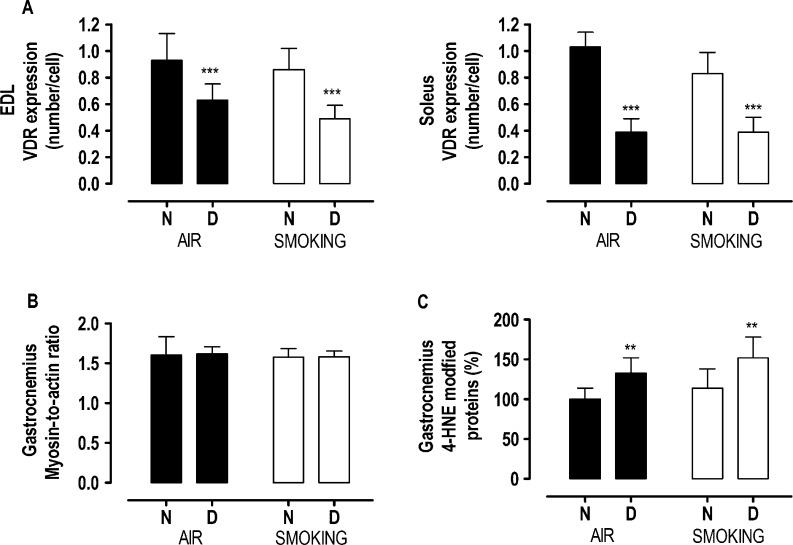
Vitamin D receptor (VDR) expression (A) in extensor digitorum longus and soleus muscles, myosin-to-actin ratio in the gastrocnemius muscle (B) and 4-hydroxy-2-nonenal modified protein (C) in the gastrocnemius muscle after 18 weeks in mice with normal (N) or deficient (D) vitamin D levels, breathing room air (black bars) or exposed to cigarette smoke (white bars). Values are mean±s.d. Values are expressed as percentage form N-air in Fig. 5C. **P<0.001 and ***P<0.0001 effect of vitamin D deficiency. There is no interaction between vitamin D deficiency and smoking.

Smoking did not affect VDR expression. By contrast, VDR expression in both EDL and soleus muscles was reduced in vitamin D-deficient mice compared with mice with normal vitamin D levels (*P*<0.0001), with no effect of smoking (Fig. 6A and Supplementary Figure 3). The reduction in VDR expression with vitamin D deficiency was more pronounced in the soleus muscle (−57%) compared with the EDL muscle (−37%; interaction term *P*<0.05).

### Myosin-to-actin ratio

At 18 weeks, myosin and actin content of the gastrocnemius muscle was similar whatever the conditions. As a consequence, neither smoking nor vitamin D deficiency had an effect on the myosin-to-actin ratio of the gastrocnemius muscle (Fig. 6B).

### Oxidative stress

In the gastrocnemius muscle, smoking increased lipid peroxidation, as shown by the 15% increase in 4HNE adduct formation (*P*=0.06). Vitamin D deficiency increased 4HNE with 33% (*P*<0.001; Fig. 6C), but there was no interaction effect between smoking and vitamin D deficiency on 4HNE adduct formation.

## Discussion

This is the first study examining the effects of vitamin D deficiency on skeletal muscle function in a smoking mouse model. In this study, we show that smoking causes early emphysema in vitamin D-deficient mice, with more pronounced lung inflammation and more tissue destruction at 18 weeks of follow-up. We also show that vitamin D deficiency and smoking exert different effects on skeletal muscles. Whereas smoking resulted in a loss of body and muscle mass and fiber atrophy, vitamin D deficiency affected muscle force of both EDL and soleus muscles. As a consequence, the combination of chronic cigarette smoke exposure and long-lasting vitamin D deficiency resulted in the poorest skeletal muscle conditions at 4.5 months of follow-up.

As expected, smoking caused the development of emphysema (Rinaldi *et al.* 2012) and lung inflammation (Vlahos *et al.* 2006, Hansen *et al.* 2013). In addition, body mass started to reduce shortly after initiation of smoking (De Paepe *et al.* 2008, Tang *et al.* 2010, Barreiro *et al.* 2012, Rinaldi *et al.* 2012, Caron *et al.* 2013, Hansen *et al.* 2013), and loss of muscle mass was present at all time points in all muscle types (EDL, soleus, and gastrocnemius muscles; De Paepe *et al.* 2008, Tang *et al.* 2010, Barreiro *et al.* 2012). We also observed atrophy of all type II fibers after 18 weeks in the EDL muscle, and from 12 weeks onward in the soleus muscle, while fiber proportions remained unaffected. This pattern of changes corresponds to what is observed in patients with moderate COPD (Hughes *et al.* 1983, Satta *et al.* 1997). Moreover, our data are in line with the increased expression of pro-atrophy-related genes, such as Atrogin-1, *Murf1,* and *Usp19*, reported in the skeletal muscle of smoking rodents (Tang *et al.* 2010, Liu *et al.* 2011, Caron *et al.* 2013). Even though muscle mass was reduced and fiber type dimensions were smaller in the smoking conditions, it did not result in muscle force impairment as both absolute and specific forces were preserved. Our data indicate that smoking mainly induced muscle wasting with no repercussion on muscle function.

Although body mass gain was lower in the vitamin D-deficient mice, mass of EDL, soleus, and gastrocnemius muscle was not reduced. This is in line with several studies carried out in vitamin D-deficient rats in which calcium and phosphorus serum levels were maintained within the normal range (Bhat *et al.* 2013, Ceglia *et al.* 2013). Interestingly, we observed a reduced contractility in both muscle types from 12 weeks onward in the vitamin D-deficient conditions. This loss of force was not related to muscle wasting, because neither muscle mass loss nor fiber atrophy was present. Moreover, muscle force was impaired in vitamin D-deficient conditions even when corrected for cross-sectional area. Until now, previous reports indicated that the detrimental effects of vitamin D deficiency on muscle force only occurred when coupled with phosphorus deficiency, because restoring phosphorus levels normalized muscle force (Schubert & DeLuca 2010). Similarly, others suggested that hypocalcemia induced by profound vitamin D deficiency either did not reduce muscle force (Schubert & DeLuca 2010) or was not the only factor responsible (Pleasure *et al.* 1979). As calcium and phosphorus serum levels were maintained within the normal range throughout the diet, the observed effects in the current study pertain to vitamin D deficiency *per se.*

We then attempted to understand how vitamin D deficiency may lead to muscle dysfunction. First, because vitamin D plays a role in regulating contractile proteins (Girgis *et al.* 2013), vitamin D deficiency might result in impaired contractility through alterations in key contractile proteins. Indeed, previous studies have shown that the content of actomyosin was reduced in vitamin D-deficient rats (Stroder 1966), as was actin in vitamin D-deficient chicks (De Boland *et al.* 1983). However, the expression of two major contractile proteins in skeletal muscles, actin and myosin, was not influenced by vitamin D serum levels, indicating that this could not explain the observed reduced muscle force in vitamin D-deficient animals.

Secondly, vitamin D deficiency is known to alter muscle contraction kinetics by reducing calcium reuptake into the sarcoplasmic reticulum, thereby leading to a prolongation of the relaxation phase of muscle contraction (Rodman & Baker 1978). As a consequence, less calcium is released from the sarcoplasmic reticulum upon a new muscle contraction. In this way, vitamin D deficiency may impair muscle force. In the current study, we indirectly assessed whether calcium reuptake was altered by measuring twitch half-relaxation time, as an index of muscle relaxation. Because twitch half-relaxation time was identical in the muscles of mice with normal and low vitamin D levels, it is unlikely that vitamin D deficiency affected muscle force by altering calcium reuptake in the sarcoplasmic reticulum.

Thirdly, through binding to its receptor (VDR), vitamin D may have an impact on muscle contraction by activating genes implicated in protein synthesis and by initiating complex second messenger pathways that influence calcium flux. Interestingly, a positive association between serum vitamin D levels and VDR concentration has been reported in skeletal muscle of animals (Domingues-Faria *et al.* 2014), in skeletal muscle cell lines (Girgis *et al.* 2014) and also in human muscle biopsies (Pojednic *et al.* 2015). As vitamin D deficiency results in downregulation of VDR expression, impairment of muscle contraction may be expected after vitamin D deficiency. In the current study, we indeed observed a downregulation of the VDR with vitamin D deficiency in both muscles, which could eventually explain the loss of muscle force with vitamin D deficiency. However, as the downregulation of VDR was more severe in the soleus muscle (−57%) compared with the EDL muscle (−37%), while the loss of muscle force was more pronounced in the EDL muscle compared with the soleus muscle, reduced VDR expression could not fully explain force impairment with vitamin D deficiency.

Finally, knowing that vitamin D or vitamin D analogs are able to limit oxidative stress in animals (Hamden *et al.* 2009, Husain *et al.* 2009, Tanaka *et al.* 2011) and keeping in mind that serum levels of vitamin D were found to be related with the activity of antioxidant enzymes in rat gastrocnemius muscle (Gonzalez-Reimers *et al.* 2010), we explored whether vitamin D deficiency may have affected muscle force in our study by enhancing muscle oxidative stress. In fact, mild oxidative stress as shown by increased protein oxidation and nitrosative stress and reduced activities of the antioxidant enzymes has been reported in the muscles of vitamin D-deficient rats (Bhat & Ismail 2015). In the current study, we assessed 4HNE as an index of lipid peroxidation. We observed an increase in 4HNE with vitamin D deficiency, indicating that vitamin D deficiency *per se *may have affected skeletal muscle function by enhancing oxidative stress. Intriguingly, because the activity of several antioxidant enzymes is higher in the type I oxidative fibers, compared with the more glycolytic type II fibers (Powers *et al.* 1994), it is suggested that the type II fibers are likely to be more vulnerable to oxidative stress. This might explain why loss of muscle force with vitamin D deficiency is higher in the EDL muscle (containing 100% type II fibers) compared with the soleus muscle (containing 63% type II fibers).

In addition to the clear interactions between smoking and vitamin D deficiency on lung inflammation and emphysema progression, a synergic effect of vitamin D deficiency with smoking was observed on the loss of soleus mass after 18 weeks. The soleus muscle might be particularly vulnerable to the combination of vitamin D deficiency and cigarette smoking due to its fiber composition. Indeed, the soleus muscle contains approximately 37% type I fibers, which are mainly affected by cigarette smoking (Montes de Oca *et al.* 2008), and 63% type II fibers, which are affected by vitamin D deficiency (Augusto *et al.* 2004, Bloemberg & Quadrilatero 2012, Ceglia & Harris 2013). Surprisingly, type II fiber atrophy was slightly more pronounced in the EDL muscle when vitamin D deficiency was combined with smoking. It is, however, important to mention that this effect was only present in the type IIa and IIx fibers, which represent about one third of all the fibers. The effect was definitely too low to affect EDL mass. Finally, vitamin D deficiency combined with smoking did not aggravate the reduction in muscle force in either muscles.

Although the current mouse model reflects a mild form of the human disease in terms of a lung disease, our data are particularly relevant for patients with COPD in whom limb muscle adaptation to COPD consists of a fiber shift toward a type II profile. Interestingly, these type II fibers seem to be preferentially affected in our model, as shown by predominant fiber atrophy with smoking, and the potentially higher vulnerability for oxidative damage. In humans, it is pertinent becuase type II fibers are the first to be recruited to prevent falling (Ceglia & Harris 2013), and falling remains a problematic concern in patients with COPD. Moreover, vitamin D deficiency has been repeatedly associated with falls and meta-analyses clearly show a beneficial effect of vitamin D supplementation on fall prevention in the general population (Bischoff-Ferrari *et al.* 2009, Murad *et al.* 2011). Our data may corroborate with these clinical findings and suggest that selective dysfunction of muscles containing predominantly type II muscle fibers may occur with vitamin D deficiency in patients with COPD.

In the current study, the serum levels of 25-(OH)D were four times lower in the vitamin D-deficient mice compared with mice with normal vitamin D levels. A similar drop in serum levels of 25-(OH)D has been previously reported in mice fed with a vitamin D-deficient diet (Crane-Godreau *et al.* 2013, Domingues-Faria *et al.* 2014). As these deficient levels are fivefold higher than what is usually targeted for studying the immunological defects, the current observations are linking skeletal muscle dysfunction to milder but long-lasting deficiencies throughout life. This is particularly relevant for the elderly COPD populations in whom moderate deficiency (10–20 ng/ml) is much more prevalent as compared with levels less than 10 ng/ml (Ramavat 1999, Paterson & Ayoub 2014) in case of rickets, which is known to present with muscle dysfunction (Torres *et al.* 1986, Veilleux *et al.* 2013).

In conclusion, although smoking mainly in general caused loss of muscle mass, moderate vitamin D deficiency was associated with muscle dysfunction in a muscle type dependent manner. Both factors clearly contribute to a worse health status of the skeletal muscle in a smoking mouse model of mild COPD.

## Supplementary data

This is linked to the online version of the paper at http://dx.doi.org/10.1530/JOE-15-0491.

## Declaration of interest

The authors declare that there is no conflict of interest that could be perceived as prejudicing the impartiality of the research reported.

## Funding

This work was supported by the Research fund of the KULeuven (OT/11/088) and AstraZeneca Pharmaceuticals.
